# Revolution

**Published:** 2004-09-15

**Authors:** Lynne S. Wilcox

After the establishment of written language, the most revolutionary development in human communication was the invention of the printing press in the 15^th^ century (1). Before then, books were handwritten, rare, and expensive. Medieval monasteries supported the transcription of new manuscripts from existing ones, and errors were common because of spelling, handwriting, and abbreviation idiosyncrasies. Because opportunities to read were few, even members of noble families were often illiterate. The transmission of most information was oral and depended on memory (1).

After the printing press was invented, its use spread rapidly through Europe. Within 50 years, eight million books were printed for multiple disciplines, including religion, law, and medicine (1). Some of the most common publications were "how-to" manuals. These instructional books, predating the *Dummies* guides by half a millennium, allowed anyone to learn new skills on topics such as herbal remedies, money management, letter writing, and cooking (2). The exchange of technical information no longer relied primarily on memory, and errors were markedly reduced (1).

But the true revolution was the societal change initiated by books. Books communicated the breadth of European culture to middle-class households, and literacy gained value among the populace. National identities developed as languages became formalized in print (2). The natural sciences advanced because scholars were able to refer to standardized concepts, definitions, texts, and images. Empirical observations were published and reviewed by other scientists and became the gold standard for scientific endeavor (1).

Another communication transformation occurred during the late 20^th^ century: the Internet. At first, during the 1970s, the Internet offered only a few military and academic applications. Much like the illiterate masses of the Middle Ages who had no books and could not read, most people 30 years ago had no access to Internet information and no skills to achieve that access. This changed by the 1990s with the convenience of personal computers, e-mail, and the Web. Today, anyone with basic computer skills can walk through the doors of a cyber café and transmit data to the other side of the globe.

This issue of *Preventing Chronic Disease* explores the application of technology to public health and chronic disease. Wagner et al describe Internet use for health information by people who have one or more of five common chronic conditions: hypertension, diabetes, cancer, heart problems, and depression (3). Block et al outline a worksite nutrition intervention program based on behavioral change models that is delivered to individuals entirely by e-mail (4). Bensley et al describe health education materials provided to special clinic populations and college students via the Web; these materials also are designed according to behavioral change models (5). Despite these opportunities, challenges arise on the "digital divide" created by differences in income, education and literacy, race and ethnicity, age, gender, geography, and disability. If we as chronic disease professionals are to use the Internet as a tool, we must rigorously examine the concept of access. Bush et al provide a thoughtful discussion on access and offer a framework that defines Internet and Web access issues for health researchers (6).

As books did, the Internet has revolutionized the way society — including government institutions — functions. Fountain describes the opportunities and challenges of digital governance as a two-stage process: first, government to citizen and second, government to government (G2G) (7,8). Many public health agencies are now passing through the first stage: providing information and services to citizens through the Internet. The more difficult tasks occur in the second stage: electronic communication among government institutions. However difficult the tasks may be, G2G exchange has the potential to drive major improvements in how government organizations interact and shape themselves.

The G2G concept is more than theory. In the early 20^th^ century, vital statistics became one of the first forms of public health data in the United States. Rothwell describes how the reengineering of state vital statistics systems will standardize information, improve accuracy, and combine data from a multitude of sources through secure Internet connections (9). A major emphasis of the reengineering is reducing error, as much a challenge in the 21^st^ century as it was in the Middle Ages. As death certificates become more accurate and timely, we can assess chronic disease mortality in more sophisticated ways and achieve more success in chronic disease prevention and health promotion.

Another example of a G2G relationship is the Public Health Information Network, which includes state, federal, and private organizations (10). This program is actually a series of interactive systems that allow rapid exchange of health information among institutions. Components include Epi-X, which delivers key health alerts — such as early signs of disease outbreaks — to appropriate professionals across the country; the National Electronic Disease Surveillance System, which creates data standards to facilitate information transfer among clinical and public health entities; and several other systems that allow secure and standardized data sharing. The use of the Internet to convey public health information is a revolution — a profound change in how public health professionals structure their data systems to make an immediate impact on public health policy.

With this issue, *Preventing Chronic Disease* completes its first year of publication. It is especially appropriate that an electronic journal marks this milestone with a discussion of health and the Internet. The 21^st^ century is still young. Today, most teenagers surf the Web with far greater skill than their grandparents, and many outperform their parents. If public health organizations follow through on their potential to use the Internet effectively, young people may become as skilled in healthy living as they are in Web use, and the greatest revolution in chronic disease prevention may arrive sooner than any of us have dared to dream.
